# Membrane Thinning and Thickening Induced by Membrane-Active Amphipathic Peptides

**DOI:** 10.3389/fcell.2016.00065

**Published:** 2016-06-24

**Authors:** Stephan L. Grage, Sergii Afonin, Sezgin Kara, Gernot Buth, Anne S. Ulrich

**Affiliations:** ^1^Karlsruhe Institute of Technology, Institute of Biological InterfacesKarlsruhe, Germany; ^2^Karlsruhe Institute of Technology, Institute of Organic ChemistryKarlsruhe, Germany; ^3^Karlsruhe Institute of Technology, Institute for Accelerator Physics and TechnologyKarlsruhe, Germany

**Keywords:** antimicrobial and cell penetrating peptides, lipid bilayer thickness, membrane thinning, magainin, gramicidin S, solid-state deuterium nuclear magnetic resonance, grazing incidence small angle X-ray scattering, oriented bilayer samples

## Abstract

Membrane thinning has been discussed as a fundamental mechanism by which antimicrobial peptides can perturb cellular membranes. To understand which factors play a role in this process, we compared several amphipathic peptides with different structures, sizes and functions in their influence on the lipid bilayer thickness. PGLa and magainin 2 from *X. laevis* were studied as typical representatives of antimicrobial cationic amphipathic α-helices. A 1:1 mixture of these peptides, which is known to possess synergistically enhanced activity, allowed us to evaluate whether and how this synergistic interaction correlates with changes in membrane thickness. Other systems investigated here include the α-helical stress-response peptide TisB from *E. coli* (which forms membrane-spanning dimers), as well as gramicidin S from *A. migulanus* (a natural antibiotic), and BP100 (designer-made antimicrobial and cell penetrating peptide). The latter two are very short, with a circular β-pleated and a compact α-helical structure, respectively. Solid-state ^2^H-NMR and grazing incidence small angle X-ray scattering (GISAXS) on oriented phospholipid bilayers were used as complementary techniques to access the hydrophobic thickness as well as the bilayer-bilayer repeat distance including the water layer in between. This way, we found that magainin 2, gramicidin S, and BP100 induced membrane thinning, as expected for amphiphilic peptides residing in the polar/apolar interface of the bilayer. PGLa, on the other hand, decreased the hydrophobic thickness only at very high peptide:lipid ratios, and did not change the bilayer-bilayer repeat distance. TisB even caused an increase in the hydrophobic thickness and repeat distance. When reconstituted as a mixture, PGLa and magainin 2 showed a moderate thinning effect which was less than that of magainin 2 alone, hence their synergistically enhanced activity does not seem to correlate with a modulation of membrane thickness. Overall, the absence of a typical thinning response in the case of PGLa, and the increase in the repeat distance and membrane thickening observed for TisB, demonstrate that the concept of peptide-induced membrane thinning cannot be generalized. Instead, these results suggest that different factors contribute to the resulting changes in membrane thickness, such as the peptide orientation in the bilayer, and/or bilayer adaptation to hydrophobic mismatch.

## Introduction

Cellular membranes have been discussed intensively as therapeutic targets. For example, many cationic amphiphilic peptides are known to preferentially bind to and destabilize bacterial membranes, endowing them with promising potential against infectious diseases. Likewise, cell penetrating peptides are being developed to carry drugs across eukaryotic cell membranes. Various concepts have been proposed for their interactions with lipid bilayers. The discussed molecular mechanisms causing membrane lysis range from pore formation to interference with the membrane via a “carpet” of peptides, or to detergent-like solubilization of the lipid bilayer (Shai, 1999; Brogden, 2005; Jenssen et al., 2006; Wimley, 2010; Li et al., 2012; Henderson and Lee, 2013). Indeed, different modes of insertion of amphiphilic peptides into the membrane have been demonstrated by determining the peptide alignment with respect to the lipid bilayer, which support all these possible modes of action. For example, helical peptides have been found to adopt discrete alignment states that depend on peptide concentration and bilayer composition. With a length of typically >20 amino acids they can even insert fully into the bilayer in an upright orientation as necessary to form pores (Huang, 2000; Bürck et al., 2008; Grage et al., 2010, 2012; Grau-Campistany et al., 2015, 2016; Strandberg and Ulrich, 2015). On the other hand, by simply binding to the bilayer surface, peptides with an amphiphilic structure can cause destabilization of membranes through membrane thinning (Huang, 2006; Khandelia et al., 2008; Grage et al., 2010; Bertelsen et al., 2012).

Many helical cationic amphiphilic peptides, such as the prototypic magainin 2, insert into the headgroup region with a helix orientation parallel to the membrane surface (Matsuzaki et al., 1994). The extra space requirement in the headgroup region due to this location leads to an expansion of the bilayer surface area. Because the membrane can be regarded as an incompressible fluid, the area expansion requires a reduction in bilayer thickness to maintain a constant volume. Such membrane thinning induced by surface-aligned amphiphilic peptides has been indeed observed experimentally (Ludtke et al., 1995; Staudegger et al., 2000; Chen et al., 2003; Mecke et al., 2005; Jang et al., 2006; Kim et al., 2009; Sun et al., 2015). The opposite response of bilayers to amphiphilic helical peptides, i.e., an increase in bilayer thickness, has also been reported in rare cases. For example, the antimicrobial peptide PGLa induces thickening of DPPG membranes (Pabst et al., 2008). All in all, it remains yet to be understood which properties of amphiphilic peptides lead to the typical reduction or to an unusual increase in membrane thickness. There are many open questions such as to what extent the peptide orientation with respect to the membrane is connected to a change in membrane thickness, or whether peptide size, hydrophobic profile or secondary structure play a role as well. Answers to these questions may give important clues on the mechanisms underlying their specific membrane-perturbing activities.

**Figure 1:**
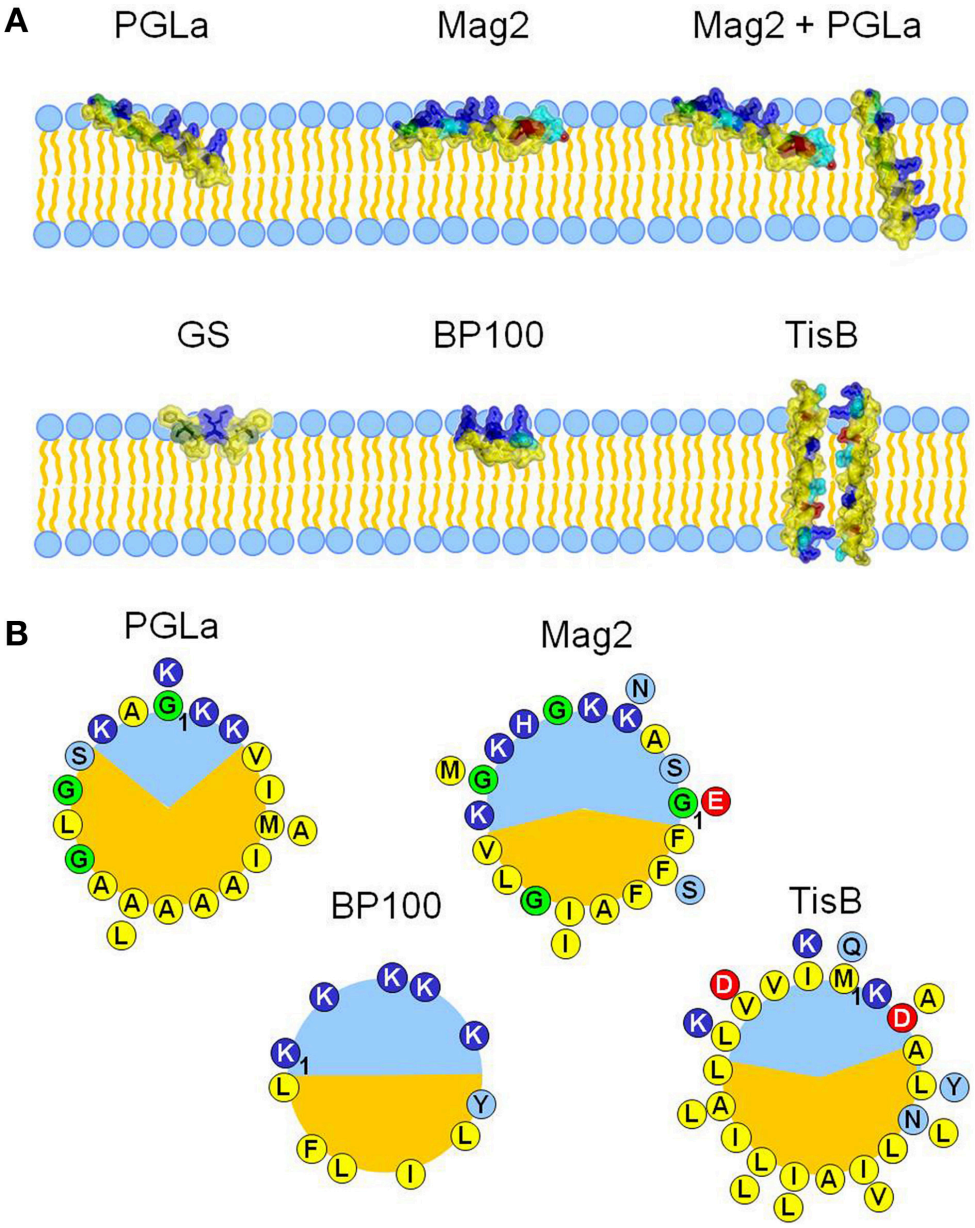
**The influence of five different amphiphilic peptides on membrane thickness was examined and compared**. All peptides are capable of interacting with cellular membranes, but they represent different structures, sizes and membrane alignments **(A)**, and different amphiphilic profiles **(B)**. PGLa and magainin 2 (Mag2) are α-helical cationic antimicrobial peptides from *X. laevis*, with lengths of 21 and 23 amino acids, respectively. They adopt an alignment on the surface of the bilayer, but PGLa is able to tilt into the membrane core at high concentrations. It can even insert fully in an upright orientation in the presence of Mag2, with which it forms a synergistically active pair. Gramicidin S (GS) from *A. migulanus* is a cyclic β-pleated decapeptide, and it aligns preferentially on the membrane surface, just like the α-helical designer-made peptide BP100 (11 amino acids). The amphiphilic α-helical peptide TisB is part of a stress-response system in *E. coli* and forms a transmembrane dimer. The different alignment states and subsequent interaction with the bilayer are in part a result of the differences in the amphiphilicity of the peptides, which are visualized for all helical peptides as helical wheels **(B)**, with charged residues drawn in red and dark blue, polar residues in light blue, glycines in green, and hydrophobic residues in yellow. PGLa possesses a larger hydrophobic sector (shown in orange) than Mag2 or BP100, correlating with its higher propensity to tilt into the membrane. TisB can dimerize in the membrane via its polar sector.

To get a better understanding of how amphiphilic peptides influence membrane thickness, we characterized and compared here the membrane interactions of five representative peptides with different sizes, secondary structures, amphiphilic profiles and resulting propensities to tilt into the bilayer (Figure 1). Prominent examples for membrane thinning are cationic amphiphilic α-helices, such as magainins. As two prototypic peptides of this peptide super-family, we studied the membrane interaction of PGLa and magainin 2 (Mag2) from the skin of *X. laevis*. PGLa is known to adopt three discrete alignment states, depending largely on peptide:lipid ratio, but also on temperature, pH, and lipid composition (Glaser et al., 2005; Afonin et al., 2008a, 2014; Misiewicz et al., 2015b). At low concentration, PGLa assumes a surface-bound state (S-state) with the helix axis parallel to the bilayer plane, but it gets tilted into an oblique alignment above a peptide:lipid threshold of ~1:80 (T-state). Mag2 also exhibits such behavior, albeit the threshold concentration of tilting is much higher than PGLa (Strandberg et al., 2013), as its smaller hydrophobic sector compared to PGLa prevents Mag2 from deeper insertion into the bilayer (Figure 1B). Interestingly, PGLa and Mag2 together exhibit a synergistically enhanced antimicrobial activity (Westerhoff et al., 1995; Strandberg et al., 2015), and PGLa is able to insert in an upright position into the membrane (I-state) in the presence of Mag2 (Tremouilhac et al., 2006; Strandberg et al., 2013). To examine a correlation between this synergy and any modulation of membrane thickness, we also studied the mixture of these two peptides. Furthermore, the influence of two much shorter (10 and 11 amino acids) amphiphilic peptides, gramicidin S (GS) and BP100, on bilayer thickness was compared with the longer helices of PGLa and Mag2 (21 and 23 residues, respectively). GS is an antimicrobial peptide from *A. migulanus* with a cyclic β-pleated structure, whose membrane interactions have been thoroughly characterized (Salgado et al., 2001; Grage et al., 2006; Berditsch et al., 2007, 2015; Afonin et al., 2008a). BP100 was originally developed as an antibacterial agent against plant microbes (Badosa et al., 2007) but is also a very effective cell penetrating agent (Eggenberger et al., 2011), forming a short regular α-helix when bound to the membrane (Torcato et al., 2013). Both GS and BP100 bind predominantly in an S-state alignment and possess a relatively high mobility as a consequence of their small size and compact shape (Salgado et al., 2001; Wadhwani et al., 2014; Zamora-Carreras et al., 2016). GS exhibits a low propensity for flipping into the membrane at high peptide:lipid ratio to form a putative oligomeric pore, which is only observed near the lipid phase transition temperature (Afonin et al., 2008b, 2014), whereas BP100 does not seem to undergo any concentration-dependent flip in its alignment (Misiewicz et al., 2015a). The behavior of these two short peptides, which are essentially located near the bilayer surface in an S-state, was contrasted in this study with yet another type of amphiphilic helical peptide. The 29mer TisB is a stress-response peptide from *E. coli*, where it occurs as part of a toxin-antitoxin regulatory system (Dörr et al., 2010). Despite its amphiphilicity, TisB has been found to span the membrane in an upright orientation, most likely as an antiparallel transmembrane dimer with the polar surfaces (see Figure 1) facing each other (Steinbrecher et al., 2012).

To assess changes in membrane thickness induced by the selected peptides in more detail, we combined two techniques in a complementary way. Using solid-state ^2^H-NMR on chain-deuterated lipids, we were able to determine the hydrophobic thickness of the bilayer, whereas grazing incidence small angle X-ray scattering (GISAXS) allowed us to measure the bilayer-bilayer repeat distance. This way, both the thickness of the hydrophobic part of the bilayer as well as the polar region including the water layer could be determined. Furthermore, both methods are compatible with oriented bilayer samples, which were used for three reasons. Firstly, this way the results of the present study can be directly related with our previous solid-state NMR measurements of the orientation of the selected peptides in mechanically oriented membranes (Salgado et al., 2001; Glaser et al., 2005; Grage et al., 2006; Tremouilhac et al., 2006; Afonin et al., 2008a,b, 2014; Strandberg et al., 2013; Wadhwani et al., 2014; Misiewicz et al., 2015a,b). Secondly, hydrated oriented samples provide a well-defined bilayer structure and morphology, with a uniform environment for the peptides. Thirdly, any broadening of the NMR signals or scattering peaks caused by anisotropy can be massively reduced, leading to enhanced sensitivity and resolution. Note, that the peptide conformation of the studied peptides has so far been found to be independent of the bilayer geometry. We can hence assume that the conformation in oriented bilayers as used here represents the functionally relevant conformation.

^2^H-NMR spectroscopy provides a simple way to deduce the hydrophobic thickness, as well as the area per lipid molecule, from the segmental order parameter S_CD, n_. This parameter describes the averaged orientation of the CD bond of the nth methylene segment with respect to the membrane normal (Saupe, 1968; de Gennes, 1974). S_CD, n_ is related to the corresponding quadrupolar splitting Δ_n_, which can be obtained directly from the ^2^H-NMR spectrum of chain-deuterated lipids, through:

$$\begin{array}{l} {\text{S}}_{\text{CD},\text{n}}=\Delta_{\text{n}}∕\Delta_{0}, \end{array}$$

where Δ_0_ = 250 kHz is the quadrupolar splitting in the absence of motion. As the order parameter is sensitive to the conformational space sampled by each chain segment, it can be used as a measure for the area that is laterally occupied by the respective chain segment. Knowing the volume of a chain segment, this area can then be converted into a contribution to the bilayer thickness arising from the considered chain segment. Quantification is possible by assuming a specific model for the distribution of chain conformations, and several procedures have been described (Schindler and Seelig, 1975; Davis, 1979; Ipsen et al., 1990; Nagle, 1993; Douliez et al., 1995; de Planque et al., 1998; Petrache et al., 2000). Following de Planque et al., and Nagle, the thickness 2 D_C_ of the acyl chain region of both bilayer leaflets is given by:

$$2\;{\text{D}}_{\text{C}}=2\;[1.27\;Å\;(\text{N-}3)\;(0.5\;+<{\text{S}}_{\text{CD}}>)]+3.75\;Å,$$

where N is the number of carbons in one chain, and < S_CD_> is the average order parameter of the chain. The area per lipid A is determined according to de Planque et al., and Nagle:

$$\text{A}=2\;[27.6\;{Å}^{2}/\;(1.27\;(0.5\;+\;{\text{S}}_{\text{p}}))],$$

where S_p_ is the average order parameter of the plateau region (carbons 2–6).

Complementary to the hydrophobic thickness 2 D_C_, the bilayer-bilayer repeat distance D_R_ in a stack of oriented bilayers is accessible from scattering experiments using GISAXS. In this grazing incidence set-up, the X-ray beam runs nearly parallel to the bilayer stack, leading to a momentum transfer nearly perpendicular to the bilayer stack in the case of small angle scattering. This way, the scattering angle θ of constructive interference encodes directly the bilayer-bilayer repeat distance D_R_, and D_R_ can be determined from the scattering geometry in a straightforward way:

$${\text{D}}_{\text{R}}=\text{m}\lambda /[\sin\theta \cos\eta +(1-\cos\theta )\sin\eta ]$$

with the wavelength λ, the order of the reflex m, and the angle between the bilayer stack and incident beam η.

We note at this point, that an evaluation of the scattering signal intensities could in principle be used to determine the form factor or electron density profile, and hence the hydrophobic thickness. We, however, chose the ^2^H-NMR approach, as this way both 2 D_C_ and D_R_ could be determined in a straightforward and direct manner from the positions of the most prominent peaks in the ^2^H-NMR spectra or scattering images, respectively.

Using the combination of solid-state ^2^H-NMR and GISAXS, we thus obtained two observables that describe hydrophobic thickness and repeat distance of the hydrated multi-bilayers. With these two values characterizing the membrane dimensions, it is possible to interpret any changes in more detail. If both 2 D_C_ and D_R_ change the same way, a genuine change in bilayer thickness can be concluded. If 2 D_C_ and D_R_ do not change simultaneously, however, this hints at some other processes beyond a simple change in membrane thickness.

## Materials and methods

### Peptides

The peptides PGLa (GMASKAGAIAGKIAKVALKAL-amide), Mag2 (GIGKFLHSA KKFGKAFVGEIMNS), BP100 (KKLFKKILKYL-amide), and TisB (MNLVDIAILILK LIVAALQLLDAVLKYLK) were synthesized using standard solid phase Fmoc protocols, and purified with reverse-phase chromatography (HPLC), as described earlier (Steinbrecher et al., 2012; Wadhwani et al., 2014; Strandberg et al., 2015). GS (cyclo-[PVOLf]_2_) was produced by fermentation of *A. migulanus*, and extracted and purified with HPLC, as outlined earlier (Berditsch et al., 2015). All peptides were at least 95% pure as determined by analytical HPLC, and were stored as lyophilized powders.

### Sample preparation

For the GISAXS experiments, all peptides were reconstituted in 1,2-dimyristoyl-*sn*-glycero-3-phosphocholine (DMPC) bilayers. For solid-state ^2^H-NMR, the peptides were reconstituted in a mixture of 80 mol% DMPC and 20 mol% chain-deuterated DMPC (DMPC-d_54_), except for samples at a peptide:lipid ratio of 1:10, for which pure DMPC-d_54_ was used. The lipids were purchased from Avanti Polar Lipids (Alabaster, AL) and used without further purification.

Oriented samples with ~10^3^ stacked bilayers were prepared on glass slides, serving as a mechanical support that leads to spontaneous orientation upon hydration. As the first step, appropriate amounts of peptide and lipid were co-dissolved in methanol to achieve the desired peptide:lipid molar ratio. Between 2 and 5 mg, or 2 mg of total material, was used for NMR or GISAXS samples, respectively. The solution was spread on 5–8 glass slides with dimensions of 12 × 7.5 × 0.06 mm (Paul Marienfeld, Germany) for NMR samples, and onto a single quartz glass slide with a dimension of 12 × 18 × 1 mm (Precision Optics, Germany) for GISAXS samples. The glass slides were dried under vacuum to remove all solvent. For NMR samples, the glass slides were stacked, and the stack was covered with a further glass slide. The NMR stack or the single GISAXS slide was hydrated by incubation in 100% relative humidity at 48°C overnight. This exposure to full humidity lead to hydrated, uniformly oriented lipid multilayers. The NMR samples were sealed with parafilm and cling foil to prevent dehydration. GISAXS samples were kept in an atmosphere of 100% relative humidity until the measurement, and they were transferred into the humidified sealed sample cell immediately prior the experiment. The quality of lipid alignment in the NMR samples was validated using ^31^P-NMR.

### Solid-state ^2^H-NMR

The solid-state ^2^H-NMR experiments were performed on a Bruker Avance III HD spectrometer equipped with a 500 MHz widebore magnet. Spectra were acquired at 76.7 MHz using either a self-built single channel probe equipped with a goniometer, or a Bruker double-tuned XH probe, both possessing a coil adjusted to the dimensions of the oriented samples. A solid-echo pulse sequence was used, with two 90° pulses of 5–6 μs and an interpulse delay of 24 μs. About 2000–10,000 scans, separated by a recycle delay of 0.5 s, were accumulated. The free induction decay was acquired for 16 ms, and processed using a window function corresponding to 100–150 Hz Lorentzian linebroadening. All measurements of samples with 20% DMPC-d_54_ were performed at 35°C, while the 100% DMPC-d_54_ samples were measured at 32°C to account for the slightly lower phase transition temperature of DMPC-d_54_. (The phase transition temperatures of DMPC and DMPC-d_54_ are ~24° and ~19°, respectively).

### GISAXS

The GISAXS experiments were performed on the SCD beamline at the ANKA synchrotron (Karlsruhe Institute of Technology, Germany). The sample was placed into a sealed Teflon sample chamber possessing Kapton entry and exit windows. The sample temperature was adjusted to 35°C using Peltier elements, and two water reservoirs assured maintenance of an atmosphere with 100% relative humidity. Temperature and humidity were controlled with a semiconductor detector placed in the sample cell (Sensirion, Saefa, Switzerland). For the scattering experiments, the sample was irradiated with an X-ray beam of 1.393 Å wavelength, and cross-sectional area of 0.5 × 5 mm^2^. Three series of acquisitions were performed with angles of incidence η of 0.05°, 0.10° and 0.15°, to cover the case of grazing incidence. The angle of incidence was adjusted by inclining the sample cell with the sample support. The scattered signal was acquired using a linear MYTHEN microstrip detector (Dectris, Switzerland), possessing 1280 pixels over 64 mm detector length, which was aligned in a direction perpendicular to the sample support. The detector was placed at a distance of 723 mm from the sample, and this way covered an angular range of 5.07°. The linear detector was moved horizontally around the vertical axis centered at the sample position, to scan an angle range from −1.44° to +1.44° in 120 steps. This scan was repeated three times with different vertical detector positions, for which the detector was rotated around the horizontal axis, which is perpendicular to the beam and centered at the sample position. In the first vertical detector position, the direct beam was 2.4° below the detector center. In the second and third vertical detector position, the detector was moved by 4.4° and 8.8°, respectively. Overall, an angular area of 2.88° horizontally and 13.87° vertically was covered.

## Results

### Hydrophobic thickness and area per lipid from solid-state ^2^H-NMR

**Figure 2:**
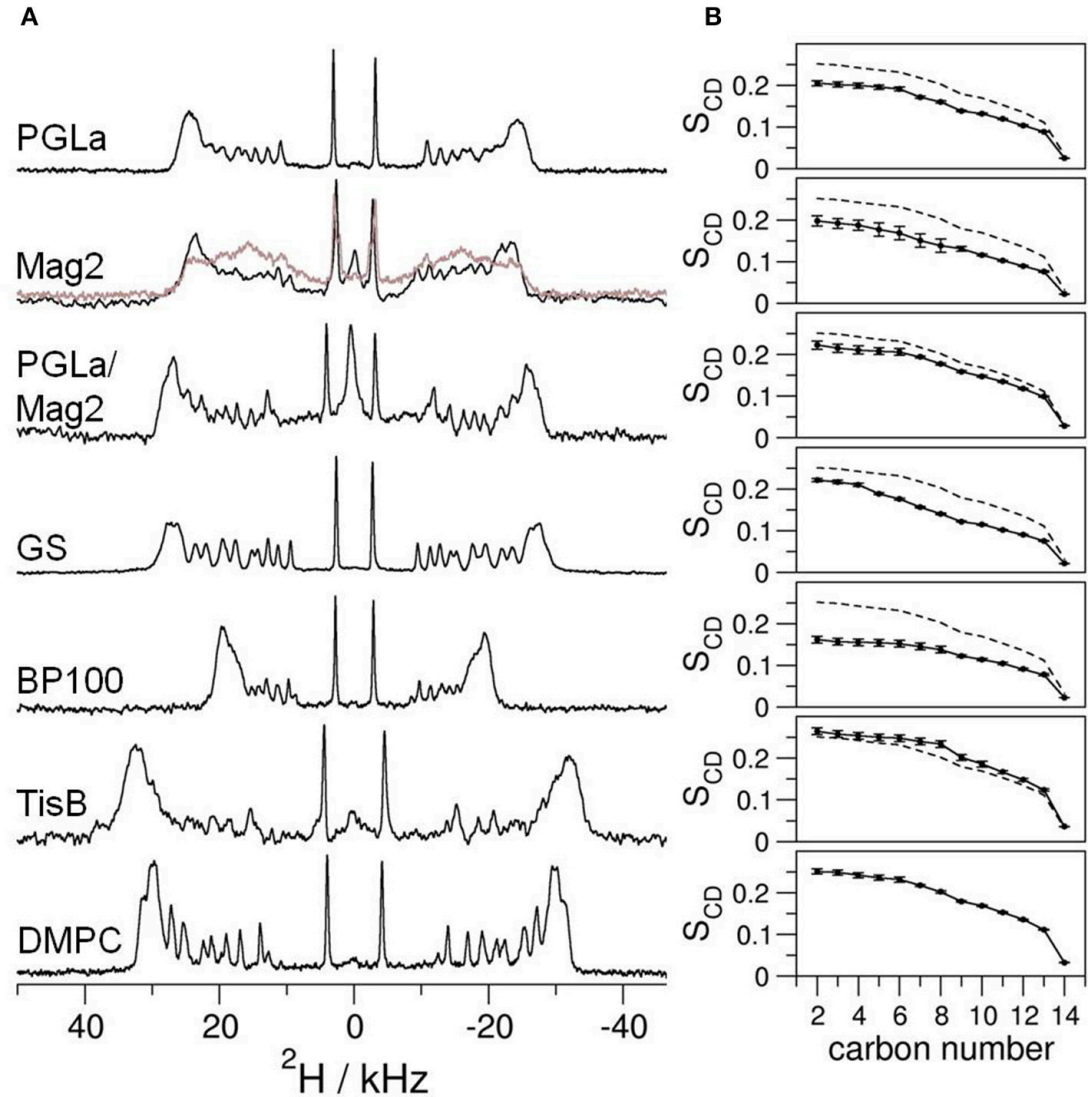
**(A)** Solid-state ^2^H-NMR spectra of oriented bilayers of chain-deuterated DMPC at 32°C. Different peptides as indicated were reconstituted into the membranes at a peptide:lipid ratio of 1:10 (mol/mol). Further peptide:lipid ratios in the range of 1:400–1:20 were studied in the same way (spectra not shown). The bottom ^2^H-NMR spectrum was obtained from plain DMPC bilayers without peptide. In some of the spectra of Mag2 at the highest peptide:lipid ratio of 1:10 we noticed two components (displayed in gray). **(B)** To assess the mobility, the order parameter S_CD_ of each deuterated methylene segment was extracted from the splitting of the respective quadrupolar doublet in the corresponding ^2^H-NMR spectrum. For comparison, the order parameter profile of the plain DMPC sample is shown as dashed line. The error of the order parameters, estimated on grounds of the linewidths of the ^2^H-NMR spectra, is indicated by error bars.

To gain insight into how amphiphilic peptides change the hydrophobic thickness of a bilayer, solid-state ^2^H-NMR spectra of DMPC with 20% chain-deuterated lipid were acquired, containing reconstituted PGLa, Mag2, PGLa/Mag2 mixture, GS, BP100, or TisB. We monitored the influence of the peptide on the bilayer as a function of peptide:lipid ratio, by measuring samples with molar ratios of 1:400, 1:200, 1:80, 1:40, 1:20, and 1:10 (exemplary ^2^H-NMR spectra for a peptide:lipid ratio of 1:10 are shown in Figure 2A). Strong variations in the spectral widths are seen in the presence of the five different peptides, indicating some obviously different interactions in these systems under comparable conditions (Figure 2A). To assess how the lipid chain mobility and the conformational distribution of the methylene segments is influenced by the peptides, the quadrupolar splittings of the ^2^H-NMR spectra were converted into an order parameter profile (see Figure 2B for examples with a peptide:lipid ratio of 1:10). In most cases, the chain order was found to be reduced compared to the lipid sample without peptide (dashed lines in Figure 2B), except for the case of TisB, where an increase in the order parameter profile was observed. The order parameters changed across the entire chain rather uniformly, but the amplitude of change varied substantially amongst the different peptides.

**Figure 3:**
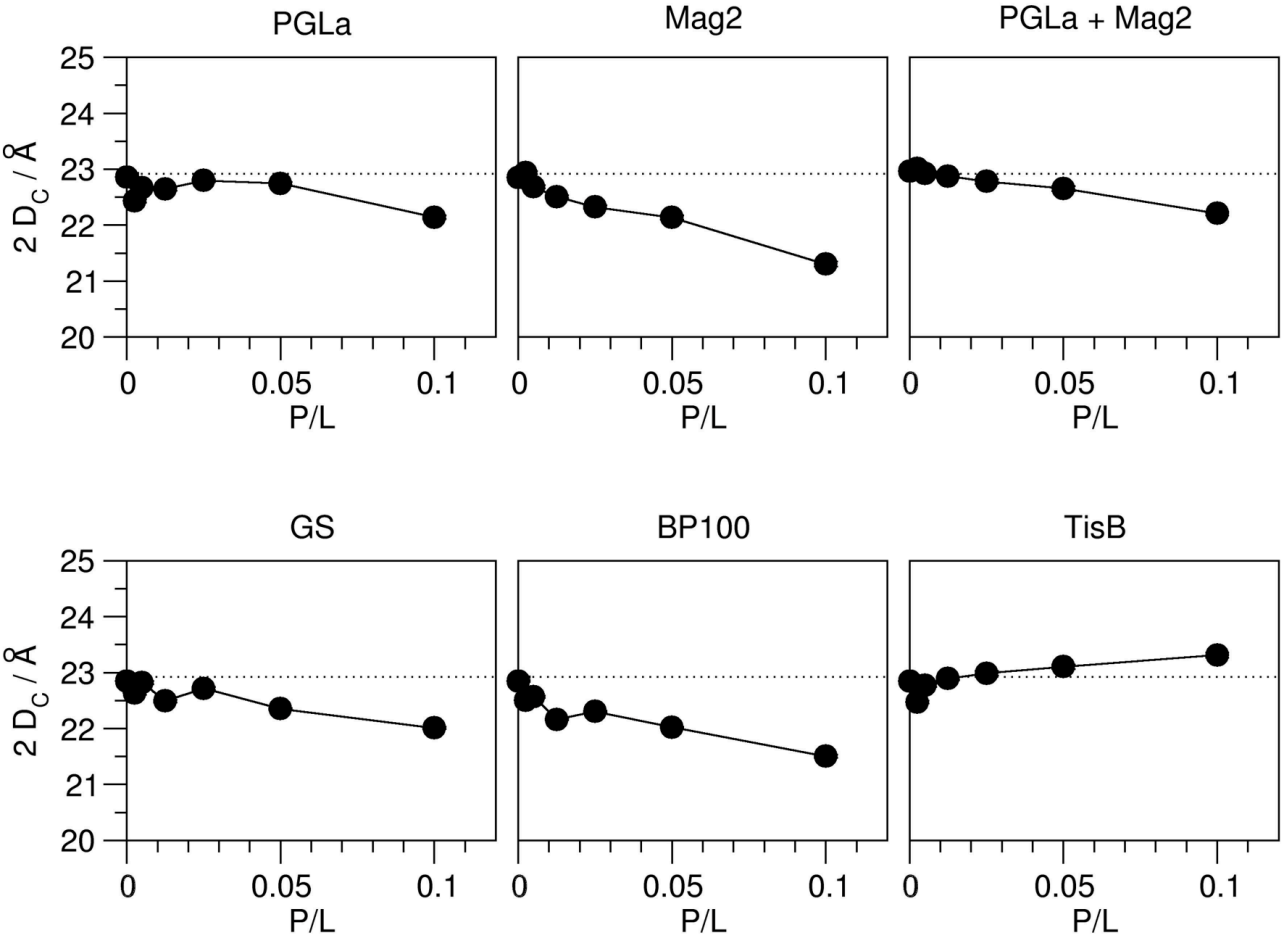
**Hydrophobic thickness 2 D_C_ of DMPC bilayers (containing 20% chain-deuterated DMPC) with reconstituted peptides as indicated, as a function of peptide:lipid ratio (P/L, mol/mol)**. The thickness was calculated from the order parameter averages, measured by ^2^H-NMR (see Figure 2). The experimental errors of the thickness values were between 0.01 and 0.05 Å, though the total errors are likely dominated by errors associated with the model assumptions underlying the thickness calculation. The dotted line indicates the hydrophobic thickness of the plain DMPC bilayer. Most peptides (Mag2, PGLa/Mag2 mixture, GS, BP100) induce a reduction of the hydrophobic thickness. PGLa showed no influence up to a peptide:lipid ratio of 1:20, and TisB caused an increase in 2 D_C_.

As outlined in the introduction, the ^2^H-NMR order parameters can be converted into the thickness of the acyl chain region of the bilayer 2 D_C_, and also into the area per lipid A, on the basis that these quantities reflect the distribution of acyl chain conformations. This way, we obtained a hydrophobic thickness of 22.9 Å for the plain DMPC bilayer, which compares well with literature values (Table 1). The addition of peptides lead to pronounced changes in this thickness, and in all cases a continuous unidirectional change with increasing peptide:lipid ratio was observed (Figure 3). Most peptides reduced the hydrophobic thickness, though to a different extent. The strongest thinning of up to ~2 Å was encountered for Mag2 and BP100, while GS and the PGLa/Mag2 mixture induced only moderate membrane thinning by ~1 Å. Notably, PGLa did not cause any change in hydrophobic thickness up to a peptide:lipid ratio of 1:20, and only at 1:10 a moderate thinning of ~1 Å was seen. Most remarkably, TisB actually increased the bilayer thickness.

**Table 1:** **Selected literature values of the hydrophobic thickness 2 D_*C*_ and area per lipid A of fluid DMPC bilayers**.

	**Method and membrane model**	**2 D_C_/Å**	**A/Å^2^**
This study	^2^H-NMR on oriented bilayers	22.9	58.9
de Planque et al., 1998	^2^H-NMR on multilamellar vesicles	22.5	61.8
Petrache et al., 2000	^2^H-NMR on multilamellar vesicles	25.2	61.4
Kučerka et al., 2005	X-ray on unilamellar vesicles and oriented bilayers	25.4	60.6
Petrache et al., 1998	X-ray on multilamellar vesicles	26.2	59.7

**Figure 4:**
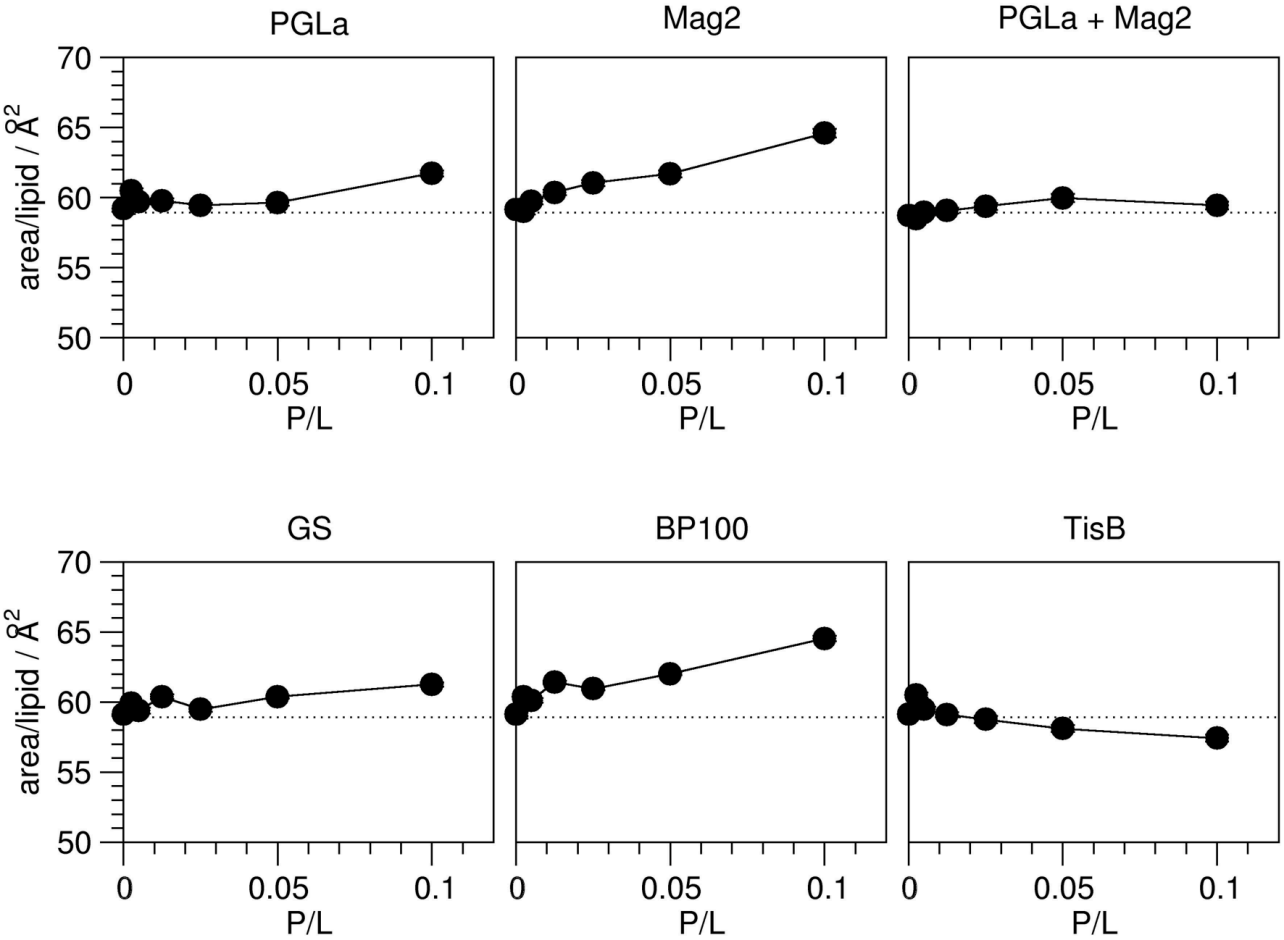
**Area per lipid in the presence of peptides as indicated, as a function of peptide:lipid ratio (P/L, mol/mol)**. The area per lipid molecule was derived from the order parameters obtained from ^2^H-NMR (Figure 2). The experimental errors of the area per lipid values were between 0.1 and 0.2 Å^2^, though the total errors are likely dominated by errors associated with the model assumptions underlying the area calculation. The dotted line indicates the area per lipid obtained for plain DMPC bilayers. Most peptides (Mag2, GS, BP100) induce an increase of the area per lipid. PGLa showed no influence up to a peptide:lipid ratio of 1:20, and also the PGLa/Mag2 mixture exhibited only a marginal increase of the area per lipid. TisB lead to a reduction of the area per lipid.

The observed peptide-induced changes in hydrophobic thickness are also reflected in corresponding modulations of the area per lipid molecule (Figure 4). For plain DMPC without any peptide, the area of 58.9 Å^2^ is close to values found in the literature (Table 1). In all cases where the addition of peptide led to a decrease in 2 D_C_, the area per lipid A increased, and *vice versa*. As for the hydrophobic thickness, the largest changes in area were found for Mag2 and BP100.

### Bilayer-bilayer repeat distance from GISAXS

To complement the hydrophobic thickness data from ^2^H-NMR, we also measured the bilayer-bilayer repeat distance D_R_ using GISAXS. This way, it was possible to address independently the polar membrane region and the inter-bilayer water layer. As in the case of hydrophobic thickness, we monitored the influence of PGLa, Mag2, a PGLa/Mag2 mixture, GS, BP100, and TisB as a function of peptide:lipid ratio, for which we prepared oriented samples with the same ratios as for ^2^H-NMR (1:400, 1:200, 1:80, 1:40, 1:20, 1:10).

**Figure 5:**
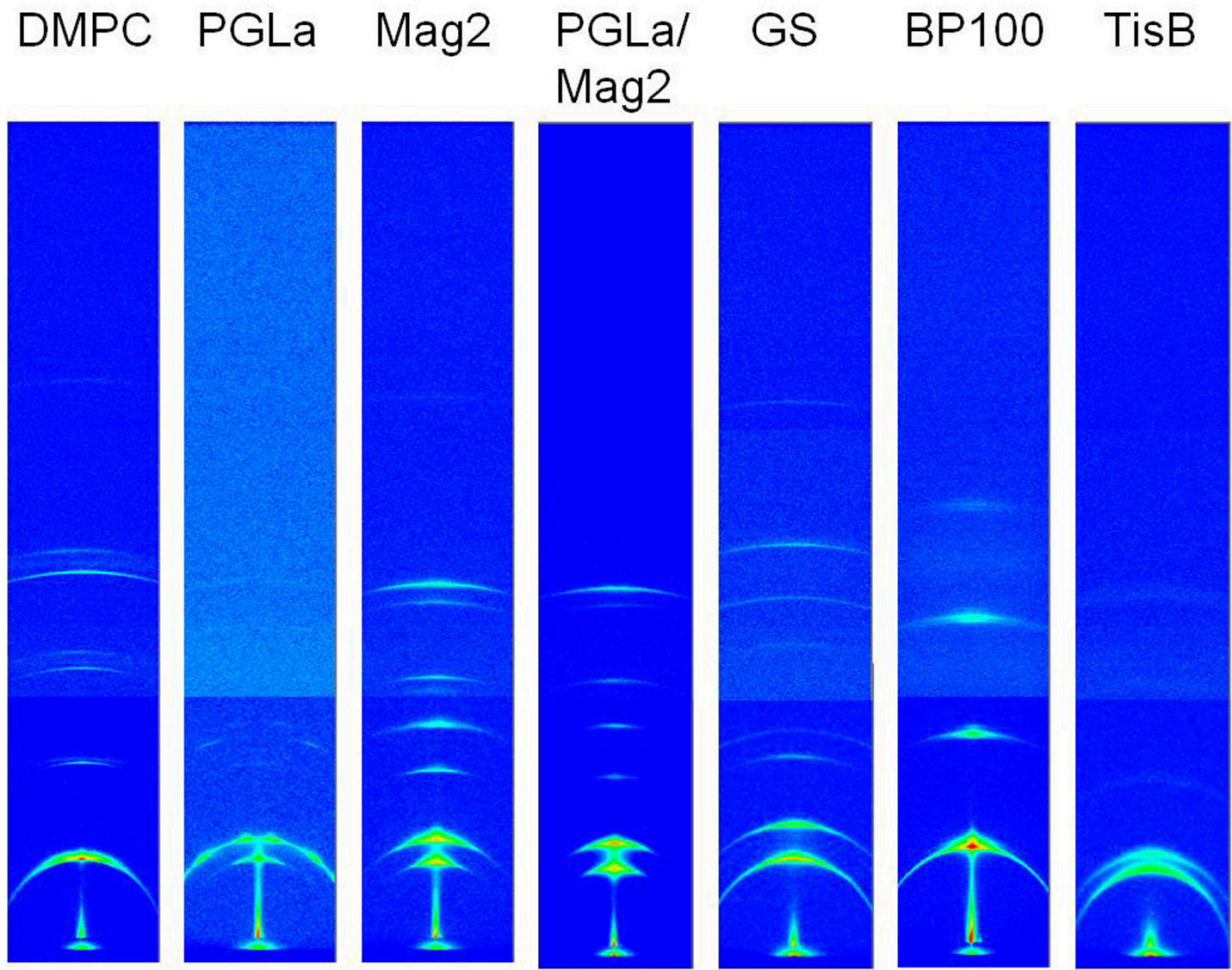
**Combined unprocessed GISAXS images covering an angular range of 2.88° horizontally and 13.87° vertically, acquired from oriented DMPC bilayers with reconstituted peptides as indicated at 35°C**. The data of a peptide:lipid ratio of 1:10 are shown here, further ratios in the range of 1:400 and 1:20 were measured the same way. The scattering image of plain DMPC bilayer stacks is shown on the left for comparison. The images are displayed such that the sample/bilayer normal runs vertically.

**Figure 6:**
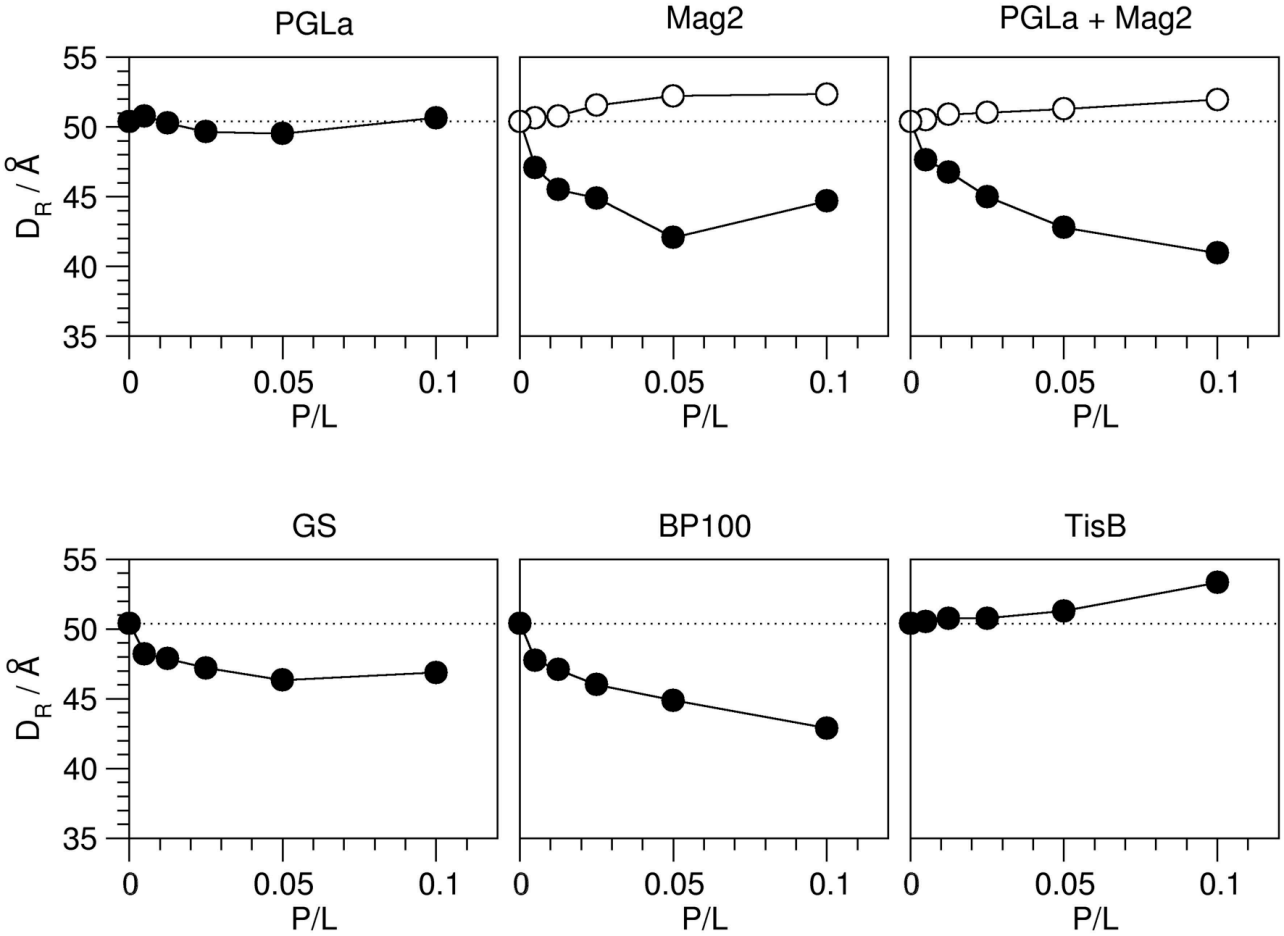
**Bilayer-bilayer repeat distance D_R_ of DMPC bilayers in the presence of the different peptides as indicated, as a function of peptide:lipid ratio (P/L, mol/mol)**. The distances were obtained from the first order reflection peaks of the respective GISAXS images, measured from oriented samples at 35°C (see Figure 5). The experimental errors of the repeat distances were estimated to be around 1–2 Å. In those cases where a second minor component was consistently present over several peptide:lipid ratios, presumably reflecting edge effects of gel phase formation, the corresponding repeat distances are shown as open circles. The D_R_ value of plain DMPC multilayers is indicated with a dotted line. Three different modes of how the amphiphilic peptides can influence bilayer thickness were observed: PGLa caused no change in D_R_. Mag2, Mag2/PGLa, GS, and BP100 decreased D_R_, while TisB increased D_R_.

The multilayer structure in the oriented membrane sample is expected to give rise to a series of equidistant scattering signals in the GISAXS images, which fall on a line in the direction perpendicular to the sample normal. From the position of these signals, D_R_ can be directly determined. Indeed, the scattering images of the oriented samples all exhibit the expected series of reflection peaks as the most prominent feature (exemplary images for a peptide:lipid ratio of 1:10 are shown in Figure 5). The signals have a crescent-like shape, which can be attributed to imperfectly aligned bilayers. A second component, corresponding to a slightly shifted set of signals, is noticeable in many samples, most prominently in samples of Mag2, PGLa/Mag2, and GS, and especially at high peptide:lipid ratios. We also noted an occasional presence of two components in the ^2^H-NMR spectra (Figure 2A, gray line), though the origin may be different. The occurrence of an additional repeat distance likely arises from differences in the scattering of bilayers in the bulk and at the surface of the stack. For example, local temperature differences could arise between the bulk part and the region of the sample near the surface. As peptides are known to lead to a broadening of the temperature range of the lipid phase transition, such a temperature gradient could lead to the formation of gel phase bilayers in the cooler part of the sample. A gel phase fraction would have a correspondingly larger membrane thickness, which is indeed what we observed for the second component (see Figure 6). It is furthermore conceivable that the effect on the width of the lipid phase transition varies from peptide to peptide. Alternatively, certain peptides may alter the ability of the bilayers to swell and maintain hydration, leading to different degrees of hydration in the bulk and near the surface. A phase separation into peptide-rich and peptide-depleted microdomains with different bilayer thicknesses, however, is unlikely. This is because such regions would have to be correlated with one another over many adjacent bilayers in order to give rise to diffraction, which seems unlikely in fluid membranes. In the following analysis, we thus focused on the major component.

The relative changes in the D_R_ values once more show a very different influence of the individual peptides (Figure 6). Without peptide, a bilayer-bilayer repeat distance of 50.4 Å was determined, which is about 16% lower than the ~60 Å reported in the literature (Kučerka et al., 2005). Similar deviations from the expected value of D_R_ have been reported for a relative humidity close to, but not reaching 100.0% (Nagle and Katsaras, 1999). This suggests that in our experiments the relative humidity was close to, but not exactly 100.0% as well, likely as a consequence of temperature gradients in the sample cell which are difficult to avoid given restrictions on X-ray transparent windows. We note, however, that our results nonetheless give a consistent picture of the peptide-induced changes. Most peptides caused a reduction in the bilayer-bilayer repeat distance compared to plain DMPC. Mag2, PGLa/Mag2, and BP100 reduced D_R_ severely by ~7–8 Å, while GS decreased D_R_ moderately by ~4 Å. PGLa induced no change in the bilayer-bilayer repeat distance, and in samples of TisB an increase of D_R_ by ~3 Å was manifest.

## Discussion

### Models for the different effects on membrane thickness

**Figure 7:**
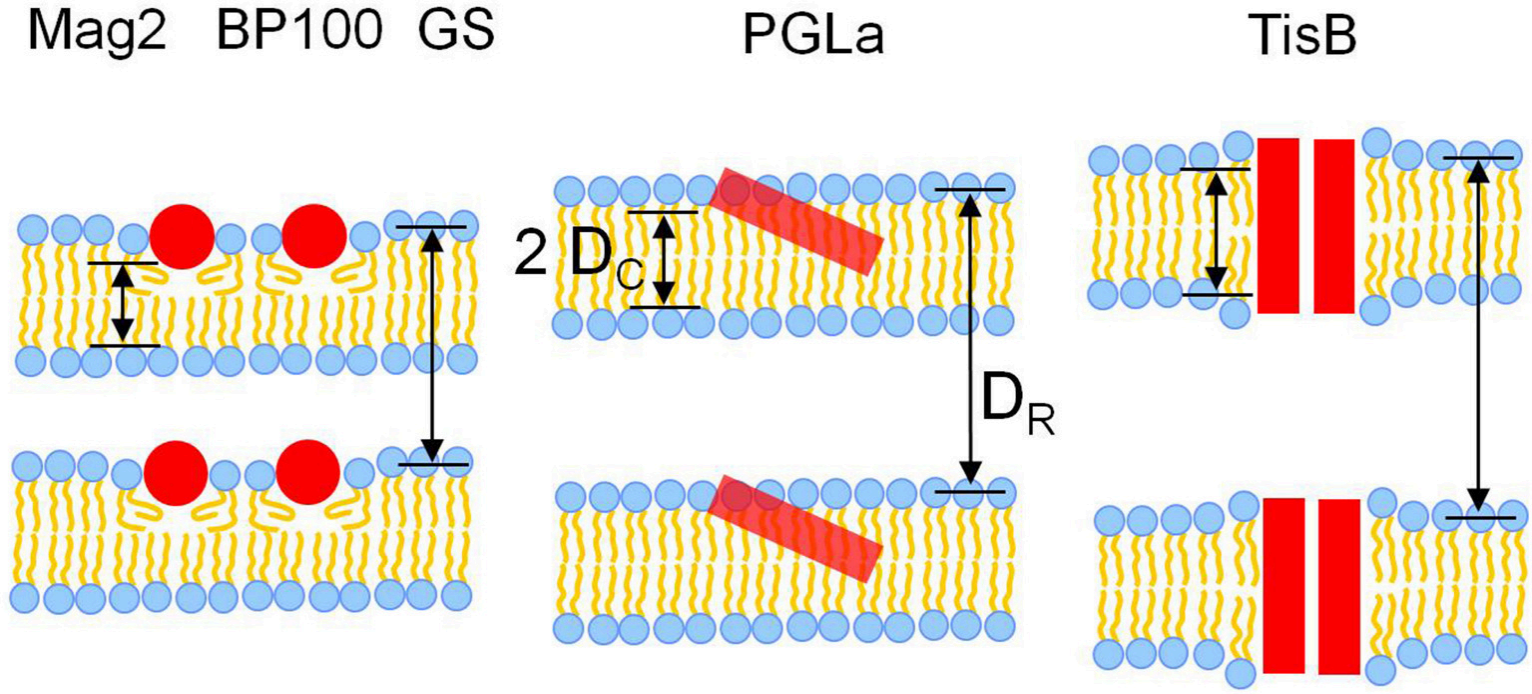
**Models for the interaction of the five different representative peptides with DMPC bilayers, illustrating the observed changes in membrane thickness**. Most peptides (Mag2, GS, BP100) caused membrane thinning, as seen from a reduction of both the hydrophobic thickness 2 D_C_ as well as the bilayer-bilayer repeat distance D_R_. PGLa induced no change in 2 D_C_ and D_R_, most likely due to its higher propensity to tilt into the membrane. TisB induced an increase of both 2 D_C_ and D_R_, which can be attributed to an adaptation of membrane thickness to the length of the transmembrane peptide dimer.

The hydrophobic thickness obtained from solid-state ^2^H-NMR and the bilayer-bilayer repeat distance from GISAXS allows us now to derive models of how different modes of bilayer interaction affect the membrane thickness. Overall, three different modes of interaction can be recognized from the data (Figure 7).

### Membrane thinning induced by Mag2, GS, and BP100

Most peptides, namely Mag2, the PGLa/Mag2 mixture, GS, and BP100 reduced both the hydrophobic thickness 2 D_C_ as well as the bilayer-bilayer repeat distance D_R_. This simultaneous decrease in both measures of thickness agrees well with the concept of membrane thinning, which has been discussed as a major mode of membrane interaction for amphiphilic peptides situated near the bilayer surface in the headgroup region. Our results also confirm, though in a different lipid system, previous experimental evidence for membrane thinning of Mag2 and GS, (Ludtke et al., 1995; Staudegger et al., 2000; Li and Salditt, 2006). Generally, the presence of the peptide in the outer bilayer regions leads to an increase in bilayer area, which in turn causes a reduction of the acyl chain layer thickness. Both, a decrease in hydrophobic thickness and an increase in the area per lipid molecule, was indeed observed for the above set of peptides. Interestingly, exactly this set of peptides consisting of Mag2, GS, and BP100 are those peptides with the lowest propensity to tilt or insert into the membrane core amongst all the peptides studied here. BP100 does not adopt a tilted alignment (Wadhwani et al., 2014; Misiewicz et al., 2015a; Zamora-Carreras et al., 2016). Mag2 can change its alignment from a surface alignment to an obliquely tilted state only at very high peptide concentrations, but it has never been reported to insert any deeper into fluid bilayers (Strandberg et al., 2013). Also gramicidin S has been found to preferentially lie flat on the bilayer surface. An insertion of GS into the membrane has only been observed in very thin bilayers of unsaturated lipids at high peptide:lipid ratios in conjunction with temperatures near the lipid phase transition (Afonin et al., 2008b, 2014). These observations support the picture that the localization of the peptide in the headgroup region leads to membrane thinning. Those peptides with a surfacial alignment are well embedded in this outer membrane layer and hence are most effective to cause membrane thinning.

### No effect of PGLa on membrane thickness

Surprisingly, despite its similarity to Mag2 in terms of structure and size, PGLa did not show any effect on membrane thickness or bilayer-bilayer repeat distance up to a peptide:lipid ratio of 1:20. Only the highest studied peptide:lipid ratio of 1:10 lead to a moderate reduction in 2 D_C_, but also here no change was observed for D_R_. The absence of thinning may be explained by the higher propensity of PGLa to adopt an obliquely tilted alignment and to insert fully into the membrane, compared to those peptides showing membrane thinning. In a tilted orientation a peptide likely displaces fewer lipids than a peptide which resides entirely in the bilayer surface region, the subsequent area increase would be lower, hence the peptide would be less effective in membrane thinning.

### Membrane thickening induced by TisB

In the presence of TisB, an increase in both hydrophobic thickness as well as bilayer-bilayer repeat thickness was observed. This effect can be readily attributed to the known transmembrane orientation of TisB (Steinbrecher et al., 2012). As a dimer, TisB presents the hydrophobic face of its amphiphilic structure toward the acyl chains (see Figure 1B). The length of the hydrophobic stretch is estimated to be 43.5Å, assuming a regular helical structure of 29 amino acids and 1.5 Å rise per residue. As the hydrophobic thickness of a fluid DMPC bilayer is only 22.8 Å, as determined in this study (or at most ~26 Å assuming literature values, see Table 1), the hydrophobic length of the TisB dimer exceeds that of the hydrophobic bilayer thickness. The observed thickening of the membrane thus seems to be a response of the bilayer to reduce this hydrophobic mismatch (de Planque et al., 1998; Holt and Killian, 2010; Grage et al., 2012; Strandberg et al., 2012).

### Relationship of changes in hydrophobic thickness and bilayer-bilayer repeat distance

In all studied peptides we observed a simultaneous increase or decrease of both the hydrophobic thickness and the bilayer-bilayer repeat distance. From this nice correlation we may conclude that the polar part of the membrane and the water layer between the bilayers are not affected by the peptide, and only the hydrophobic thickness is influenced. However, quantitatively the changes in 2 D_C_ do not match the changes in D_R_. While changes in the repeat distance D_R_ amount up to ~8 Å, the hydrophobic thickness 2 D_C_ is influenced much less, only up to ~2 Å. Nonetheless, this discrepancy could be explained by the fact that the hydrophobic thickness measured by NMR reflects an average over time and over the entire ensemble in the sample. The repeat distance, on the other hand, reflects how close adjacent bilayers can approach each other, for which the maximal extensions of the membrane, including bilayer undulations, are important. E.g., a local increase in hydrophobic thickness in only a few points, or deviations from a flat bilayer, could lead to an overall increased repeat distance. Hence a large change in the bilayer-bilayer repeat distance could be caused by a locally large but on average small change in the hydrophobic thickness, or even by local bilayer deformations not involving a change in hydrophobic thickness at all. The apparently larger influence of the peptides on the repeat distance compared to the hydrophobic thickness therefore does not necessarily indicate changes in the width of the hydration layer or of the polar regions of the bilayer.

### Synergistic enhancement of membrane thinning?

In the mixture of PGLa and Mag2 we did not observe any synergistic enhancement compared to the individual effects on bilayer thickness. The reduction in 2 D_C_ of the PGLa/Mag2 mixture is only approximately half of the reduction observed for Mag2 alone, when comparing samples with the same total pepide:lipid ratio (Figure 3). As PGLa had no effect, this finding is readily explained by a simple addition of the thinning effect of the two peptides.

However, the bilayer-bilayer repeat distance of PGLa/Mag2 changed more than expected for a mere addition. Here, D_R_ as a function of total peptide:lipid ratio of the mixture compares well with D_R_ of the pure Mag2 samples at the respective peptide:lipid ratios (Figure 6). Hence, on one hand the synergistic activity enhancement does not seem to be related to a different membrane thinning effect of the mixture compared to the individual peptides. But, on the other hand, the observed difference in response of the repeat distance D_R_ could indicate a different influence of the peptides on the headgroup region or the inter-membrane water layer, depending if they occur as a mixture or individually. For example, PGLa and Mag2, when together, might lead to a different degree of hydration compared to the individual peptides.

## Conclusions

In this study we used two complementary techniques, solid-state ^2^H-NMR and GISAXS, both on oriented membranes to get insight into the influence of amphiphilic membrane-active peptides on bilayer thickness. Two parameters could be derived by this approach in a straightforward manner: the hydrophobic thickness of the membrane, and the bilayer-bilayer repeat distance. We observed a strong correlation between the alignment states of the peptides, and their ability to modulate these two aspects of membrane thickness. Peptides which reside predominantly in the outer layer of the membrane, being embedded in the headgroup region parallel to the membrane surface (S-state), were found to cause membrane thinning. Peptides with a high propensity to penetrate more deeply and subsequently tilt into the membrane (T-state), did not influence the membrane thickness. Transmembrane peptides (I-state), with a length exceeding the bilayer hydrophobic thickness caused membrane thickening. These three different modes of influence on the membrane thickness can be understood from the location of the peptides in the membrane. When embedded in the polar/apolar interface region, the peptide leads to an expansion of the bilayer surface area, which in turn is linked to a reduction in hydrophobic thickness to maintain the volume of the lipid acyl chains. In the T-state, the interaction interface of the peptide with the outer membrane layer is reduced, hence membrane thinning is less effective. Finally, the membrane thickening observed in the fully inserted transmembrane dimer can be explained by an adjustment of the acyl chains to relax the hydrophobic mismatch between the hydrophobic peptide length and the effective bilayer thickness. Though many of the discussed aspects of peptide/lipid interaction have been studied previously, this comparative study adds a more global perspective and provides some general clues on the membrane interactions of amphipathic peptides.

## Author contributions

SG designed the experiments, prepared the samples, conducted the experiments, analyzed the data and wrote the manuscript. SA participated in the design and conductance of the experiments and sample preparation, as well as data analysis and writing of the manuscript. SK participated in the conductance of the experiments and sample preparation and discussion of the manuscript. GB was setting up the scattering experiments and participated in the design, conductance, data analysis and discussion of the scattering experiments. AU participated in design and discussion of the experiments and writing of the manuscript.

## Funding

We acknowledge funding by the Helmholtz Association (Program BIFTM).

### Conflict of interest statement

The authors declare that the research was conducted in the absence of any commercial or financial relationships that could be construed as a potential conflict of interest.
